# Invasive Versus Medical Management in Patients With Prior Coronary Artery Bypass Surgery With a Non-ST Segment Elevation Acute Coronary Syndrome: A Pilot Randomized Controlled Trial

**DOI:** 10.1161/CIRCINTERVENTIONS.119.007830

**Published:** 2019-08-16

**Authors:** Matthew M.Y. Lee, Mark C. Petrie, Paul Rocchiccioli, Joanne Simpson, Colette E. Jackson, David S. Corcoran, Kenneth Mangion, Ammani Brown, Pio Cialdella, Novalia P. Sidik, Margaret B. McEntegart, Aadil Shaukat, Alan P. Rae, Stuart H.M. Hood, Eileen E. Peat, Iain N. Findlay, Clare L. Murphy, Alistair J. Cormack, Nikolay B. Bukov, Kanarath P. Balachandran, Keith G. Oldroyd, Ian Ford, Olivia Wu, Alex McConnachie, Sarah J.E. Barry, Colin Berry

**Affiliations:** 1 Department of Cardiology, West of Scotland Heart and Lung Centre, Golden Jubilee National Hospital, Glasgow, United Kingdom (M.M.Y.L., M.C.P., P.R., J.S., C.E.J., D.S.C., K.M., P.C., N.P.S., M.B.M., A.S., A.P.R., S.H.M.H., E.E.P., K.G.O., C.B.).; 2 British Heart Foundation Glasgow Cardiovascular Research Centre, Institute of Cardiovascular and Medical Sciences (M.M.Y.L., M.C.P., P.R., J.S., C.E.J., D.S.C., K.M., A.B., M.B.M., A.S., A.P.R., C.B.), University of Glasgow, United Kingdom.; 3 Robertson Centre for Biostatistics (I.F., A.M.), University of Glasgow, United Kingdom.; 4 Health Economics and Health Technology Assessment (O.W.), University of Glasgow, United Kingdom.; 5 Department of Cardiology, Western Infirmary, Glasgow, United Kingdom (M.M.Y.L., A.B., M.B.M., C.B.).; 6 Department of Cardiology, Royal Alexandra Hospital, Paisley, United Kingdom (M.M.Y.L., S.H.M.H., E.E.P., I.N.F., C.L.M., A.J.C.).; 7 Department of Cardiology, Glasgow Royal Infirmary, United Kingdom (M.C.P., P.R., A.S., A.P.R., M.M.Y.L.).; 8 Department of Cardiology, Royal Blackburn Hospital, United Kingdom (N.B.B., K.P.B.).; 9 Department of Mathematics and Statistics, University of Strathclyde, United Kingdom (S.J.E.B.).

**Keywords:** acute coronary syndrome, clinical trial, coronary angiography, coronary artery bypass surgery, myocardial infarction, percutaneous coronary intervention

## Abstract

The benefits of routine invasive management in patients with prior coronary artery bypass grafts presenting with non-ST elevation acute coronary syndromes are uncertain because these patients were excluded from pivotal trials.

**Methods:**

In a multicenter trial, non-ST elevation acute coronary syndromes patients with prior coronary artery bypass graft were prospectively screened in 4 acute hospitals. Medically stabilized patients were randomized to invasive management (invasive group) or noninvasive management (medical group). The primary outcome was adherence with the randomized strategy by 30 days. A blinded, independent Clinical Event Committee adjudicated predefined composite outcomes for efficacy (all-cause mortality, rehospitalization for refractory ischemia/angina, myocardial infarction, hospitalization because of heart failure) and safety (major bleeding, stroke, procedure-related myocardial infarction, and worsening renal function).

**Results:**

Two hundred seventeen patients were screened and 60 (mean±SD age, 71±9 years, 72% male) were randomized (invasive group, n=31; medical group, n=29). One-third (n=10) of the participants in the invasive group initially received percutaneous coronary intervention. In the medical group, 1 participant crossed over to invasive management on day 30 but percutaneous coronary intervention was not performed. During 2-years’ follow-up (median [interquartile range], 744 [570–853] days), the composite outcome for efficacy occurred in 13 (42%) subjects in the invasive group and 13 (45%) subjects in the medical group. The composite safety outcome occurred in 8 (26%) subjects in the invasive group and 9 (31%) subjects in the medical group. An efficacy or safety outcome occurred in 17 (55%) subjects in the invasive group and 16 (55%) subjects in the medical group. Health status (EuroQol 5 Dimensions) and angina class in each group were similar at 12 months.

**Conclusions:**

More than half of the population experienced a serious adverse event. An initial noninvasive management strategy is feasible. A substantive health outcomes trial of invasive versus noninvasive management in non-ST elevation acute coronary syndromes patients with prior coronary artery bypass grafts appears warranted.

**Clinical Trial Registration:**

URL: https://www.clinicaltrials.gov. Unique identifier: NCT01895751.

## Abstract 2

Supplemental Digital Content is available in the text.

**WHAT IS KNOWN**

- There is an evidence gap on the safety and efficacy of invasive management in patients with a prior coronary artery bypass graft because they were excluded from several clinical trials of routine invasive management versus conservative management.

**WHAT THE STUDY ADDS**

- In a randomized, multicenter trial, we obtained proof-of-concept information on feasibility, efficacy, and safety of routine medical management compared with invasive management in medically stabilized patients following an acute non-ST segment elevation acute coronary syndrome and a history of prior coronary artery bypass grafts.
- Health outcomes and quality of life during a median of over 2-years follow-up were similar for patients in each group.
- In the invasive group, percutaneous coronary intervention was performed in one-third of the participants while in the medical group, only 1 (3.4%) participant crossed over to invasive management on day 30 but percutaneous coronary intervention was not performed.
- A comparative effectiveness trial involving contemporary invasive and medical therapies seems justified.

Based on the results of 10 randomized trials of invasive versus conservative medical management in patients with a non-ST segment elevation acute coronary syndrome (NSTE-ACS^1,2^; Table 1),^3–18^ invasive management is associated with a Class 1 practice guideline recommendation (Level of Evidence A).^19–21^ Around 1 in 10 patients admitted to hospital with an acute NSTE-ACS have a history of prior coronary artery bypass graft surgery (CABG).^19–22^ CABG is a standard of care for patients with obstructive coronary artery disease; however, reflecting the natural history of saphenous vein graft disease, graft occlusion is common within 10 years of surgery.^23–25^ Patients with prior CABG have a progressive longer-term risk of recurrent ischemia, including angina (>6% at 1 year),^26^ myocardial infarction (MI; >7% after 6 years,^27^ or >10% within 10 years),^28^ hospitalization for heart failure (HF; 2% within 30 days),^29^ and death (>2% at 1 year^30,31^ rising to >4% to 9% after 5 years).^22,27,32,33^ This group of patients presents a challenge to healthcare providers globally not least because of their elderly age and multimorbidity.

**Table 1.**
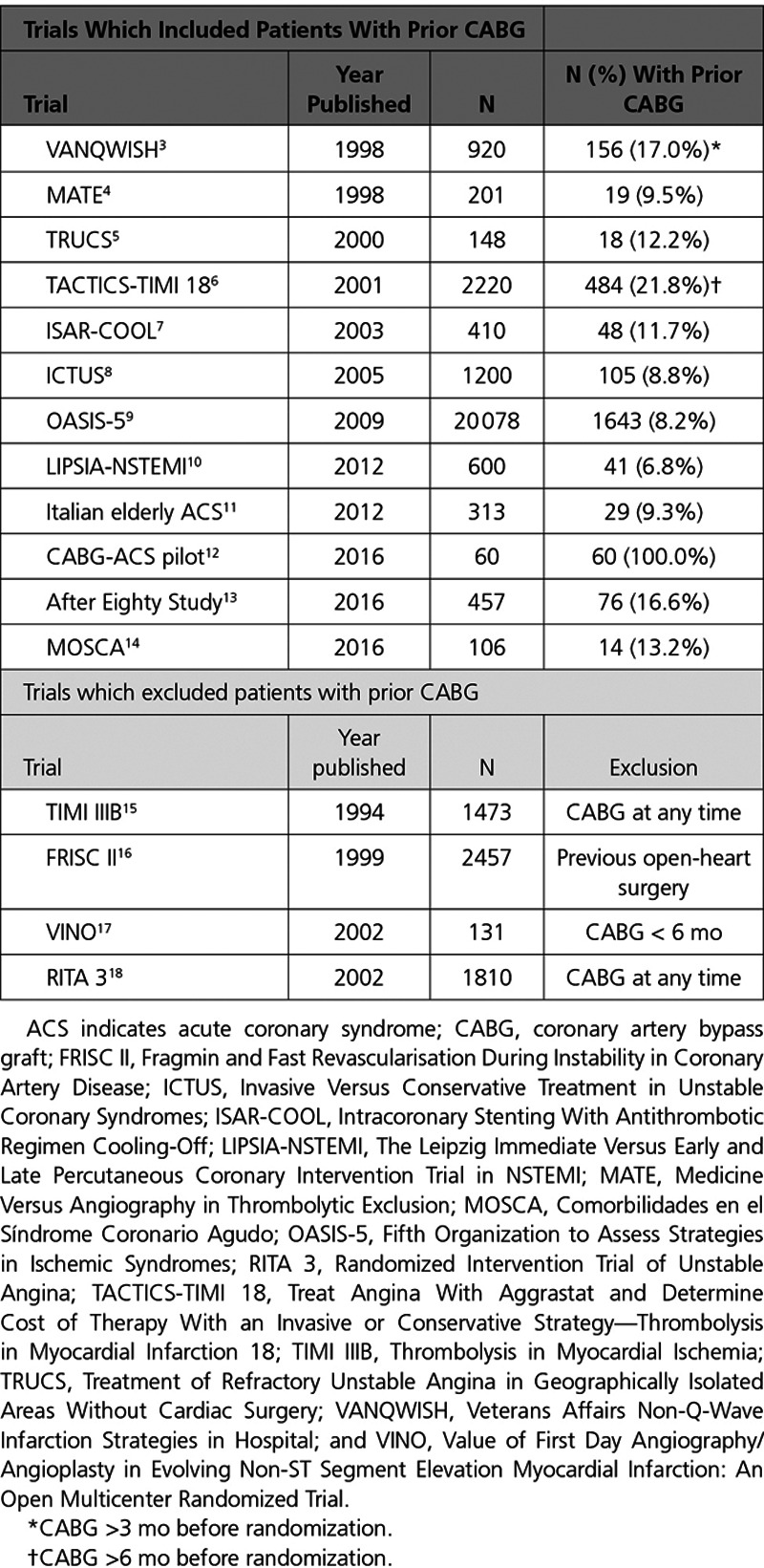
Trials of Patients With Non-ST Elevation Acute Coronary Syndromes

Some of the pivotal trials of invasive versus conservative management in NSTE-ACS, including thrombolysis in myocardial ischemia,^15^ FRagmin and Fast Revascularisation during InStability in Coronary artery disease,^34^ and RITA 3 (Randomized Intervention Trial of unstable Angina),^18^ excluded patients with prior CABG (Table 1). Therefore, the relevance of practice guideline recommendations^19,20,35^ and balance of risks and benefits in this large subgroup of patients is less certain.^36–38^ When invasive management is performed, revascularization with either percutaneous coronary intervention (PCI) or redo CABG is less likely in NSTE-ACS patients with prior CABG.^37–40^ However, advances in percutaneous revascularization techniques create new therapeutic possibilities for this patient group.

A substantive, health outcome trial of invasive management involving contemporary techniques versus noninvasive management appears warranted. However, critical uncertainties relating to the feasibility of enrollment, adherence to the randomized strategy, and overall safety undermine the rationale for such a trial.

In this study, we aimed to assess the feasibility and safety of routine noninvasive versus invasive management in patients with NSTE-ACS and prior CABG in a multicenter setting. To address this aim, we undertook a randomized, controlled, pilot trial of routine invasive management (standard of care) versus noninvasive medical management. The primary hypothesis was that in patients with NSTE-ACS and prior CABG randomization to medical management is routinely feasible, as reflected by adherence to this strategy by 30 days. Evidence of efficacy and safety in the longer term was prospectively assessed. The efficacy of each strategy was assessed by blinded assessment of all-cause mortality, nonfatal MI, or hospitalization for HF events during longer-term follow-up. The safety of each strategy was assessed by comparison of bleeding (Bleeding Academic Research Consortium types 2–4),^41^ stroke, procedure-related MI (Type 4a, European Society of Cardiology Universal Definition of MI),^42^ and worsening renal function or hemodialysis events during the index hospitalization.

## Methods

The data that support the findings of this study are available from the corresponding author on reasonable request.

### Study Design and Setting

The design of this pilot trial has been previously described.^12^ The participants were enrolled in 4 acute hospitals in the National Health Service (NHS), United Kingdom, including 2 large urban hospitals (Western Infirmary and Royal Infirmary, Glasgow) and 2 regional hospitals (Royal Alexandra Hospital, Paisley and Royal Blackburn Hospital). More details about these hospitals are detailed in the Data Supplement.

### Population

Eligibility for randomization in the trial was based on the following criteria:

#### Inclusion

(1) Unstable angina or non-ST segment elevation MI; (2) stabilized symptoms without recurrent chest pain or intravenous therapy for 12 hours; (3) prior CABG.

#### Exclusion

(1) Refractory ischemia (ie, recurrent angina with minimal exertion or at rest [ie, Canadian Cardiovascular Society class III or IV] not controlled by medical therapy); (2) cardiogenic shock; (3) lack of informed consent; (4) unsuitable for invasive management.

Patients who fulfilled the eligibility criteria were provided with an information sheet as soon as feasible after hospital admission and before referral for coronary angiography. Written informed consent was required for participation in the trial.

### Screening

The clinical research team on each site screened patients who had been hospitalized during unscheduled emergency care. Patients who were 18 years and older, of either sex, and who had a history of NSTE-ACS and prior CABG were prospectively identified. Trial participation required that the attending physician confirm there was equipoise for the potential benefits of either invasive management or noninvasive management. If either the physician or the patient did not agree, then the patient was designated as a screen failure. Informed consent for participation in the follow-up registry was then invited. Each patient was assigned a unique study number and then entered into a screening log. The community health index or NHS number was recorded to enable electronic record linkage using routinely collected NHS datasets.

### Randomization

Patients who fulfilled the inclusion criteria and did not have any exclusion criteria and who also had provided written informed consent were enrolled into the trial. Randomization was performed using an interactive voice recognition system managed by the Glasgow Clinical Trials Unit. Participants were randomized 1:1 to either the invasive group or medical group. Randomization was stratified by center, using randomized permuted blocks of length 4 and 6, with block lengths chosen at random.

### Medical Therapy

Optimal medical therapy was recommended for participants in both of the randomized groups. Guidance on uptitration of medical therapy in both groups was provided in an investigator guideline. Medical therapy included dual antiplatelet, antithrombotic, and antianginal therapies as per local protocols and international guidelines.^19,20^

### Medical Group

According to the trial protocol, study participants who had been randomized to the medical group, that is, noninvasive management, could be referred for invasive management if prespecified criteria (Data Supplement) occurred post-randomization.

### Invasive Group

Invasive management was performed early (ie, ≤72 hours wherever possible) after hospital admission. Invasive management included native coronary and bypass graft angiography and coronary and graft revascularization with percutaneous coronary intervention and CABG, as clinically appropriate.

### Screen Failures

**Figure 1.**
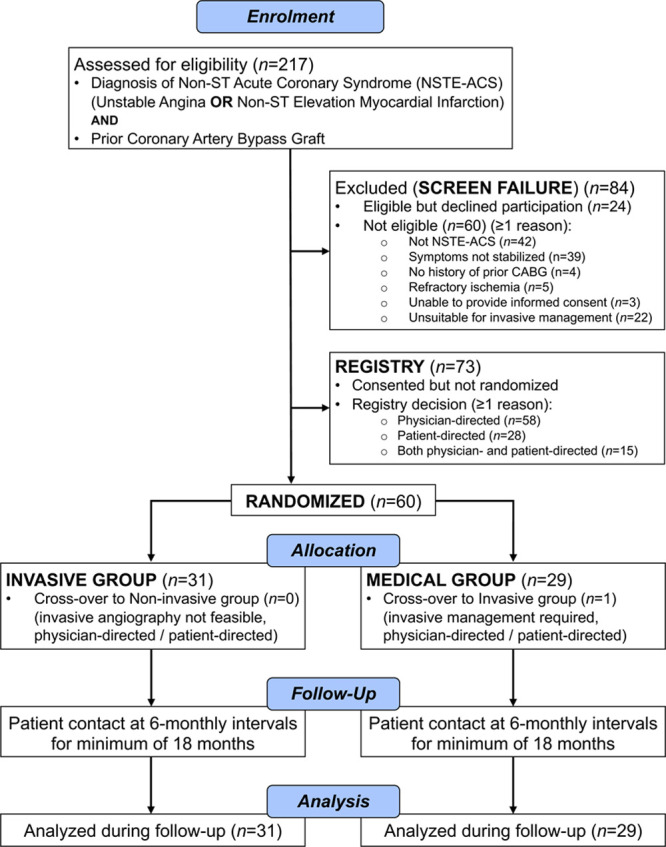
**CONSORT flow diagram.** CABG indicates coronary artery bypass graft.

Screened patients who were (1) eligible but did not consent to participate in the randomized trial or (2) did not meet eligibility criteria (Figure 1; CONSORT [Consolidated Standards of Reporting Trials] diagram) were included in a screen failure log.

### Registry

The reasons for nonparticipation of patients who were eligible for randomization were prospectively recorded: physician preference, patient preference, or both (Figure 1; CONSORT diagram).

### Follow-Up

Clinical research nurses and physicians who were independent of the study teams and aware of the group allocations conducted the follow-up assessments. They prospectively gathered information on screening, recruitment, randomization (to medical therapy or invasive management), crossover rates, and serious adverse events in patients with prior CABG and a recent NSTE-ACS.

### Sample Size

The sample size of 60 randomized participants was predetermined to be sufficient to provide information on the feasibility of randomization in a consecutive series of patients with NSTE-ACS and prior CABG who had been prospectively enrolled, ad hoc, during unscheduled care. The sample size was also intended to be sufficient to provide information on adherence with the allocated strategy within the first 30 days. The trial was not powered to assess for between-group differences in the rates of the serious adverse events contributing to the prespecified efficacy and safety outcomes.

### Outcomes

Serious adverse events during the index admission and follow-up were detected by contacting the participants at 6- and 12 months following enrollment, by reviewing medical records obtained during usual care, and routinely collected electronic health databases, including the community health index number and NHS number. The occurrence of these outcomes was prospectively entered into an electronic case report form.

#### Primary Outcome

The primary outcome was the postrandomization rate of major adverse events (coprimary composite outcome), including 1 composite outcome for efficacy and 1 composite outcome for safety. The comparison between the incidences of each outcome according to treatment group assessed the between-group difference in the proportion of major adverse events in patients allocated to noninvasive conservative management compared with invasive management.

#### Primary Efficacy Outcome

Defined as all-cause mortality, rehospitalization for refractory ischemia/angina, MI, or hospitalization for HF. The end points were assessed during the study until the final randomized patient had completed 18 months follow-up.

#### Primary Safety Outcome

Defined as bleeding (bleeding academic research consortium types 2–4),^41^ stroke, procedure-related MI (Type 4a, European Society of Cardiology Universal Definition of MI),^42^ worsening renal function, or hemodialysis during the index hospitalization.

#### Secondary Outcomes

1. Quality of life
  EuroQol 5 Dimensions 5 Levels and EuroQol Visual Analogue Scale were assessed at baseline and 6 monthly intervals for a minimum of 18 months.
2. Canadian Cardiovascular Society angina class
3. Hospitalization for refractory ischemia
  The definition of refractory ischemia is detailed in the Data Supplement.
4. Invasive management during follow-up
5. Coronary and bypass graft intervention during follow-up

### Clinical Event Committee

An independent Clinical Event Committee (CEC) reviewed serious adverse events that potentially fulfilled the definition of a primary outcome event. The CEC was blinded to all information relating to the randomization group. The CEC reviewed cases of interest to determine if they met the criteria defined in the prespecified charter. Causality assessments were not made by the CEC. The CEC included 4 cardiovascular physicians with expertise in the diagnosis and treatment of cardiovascular disorders and in the medical aspects of clinical trials. The CEC included a Chair (M.C. Petrie) and a coordinator (M.M.Y. Lee) to assist with preparation of de-identified source clinical data, reports, and communication with the clinical trials unit. The CEC followed a predetermined adjudication charter.

### Definitions of Adverse Events

The adverse events of death, procedure-related MI, stroke, major bleeding, and worsening renal function are defined as detailed in the Data Supplement.

### Follow-Up and Timing of Outcome Evaluations

Follow-up (via telephone contact, clinic visits, letter) with completion of quality of life assessments (EuroQol 5 Dimensions 5 Levels and EuroQol Visual Analogue Scale ) was maintained at 6 monthly intervals until a minimum of 18 months follow-up had been reached for the final recruited patient. Consent was obtained for long-term follow-up analyses.

Following randomization, clinical assessments involved gathering information from the standard of care clinical reviews (end of hospitalization, 30–42 days, and 1 year) and also from clinical contacts recorded in the patients’ medical records. In West of Scotland hospitals, a single system of electronic patient records is used for all hospital attendances and correspondence with primary care.

### Crossover

A crossover from 1 randomized group to another was predefined as a change of treatment strategy from invasive to noninvasive management or vice versa within 30 days of randomization. While the intention-to-treat in each group was either with noninvasive or invasive management, all treatment options remained available according to patient and physician preference, that is, patients initially randomized to medical therapy could have undergone invasive management and vice versa. No additional interventions were proposed nor were procedures withdrawn that would be needed on clinical grounds.

### Data Management and Biostatistics

The Robertson Centre for Biostatistics acted as an independent coordinating center for randomization, data management, and statistical analyses. The Centre is part of the registered Glasgow Clinical Trials Unit (National Institute for Health Research Registration number: 16). The Chief Investigator (Dr Berry) had full access to all the data in the study and takes responsibility for its integrity and the data analysis

### Statistical Analysis

Baseline characteristics of the randomized participants were summarized by group using mean (SD), or median (lower quartile, upper quartile for skewed data) for continuous variables and count (%) for categorical variables. Numbers of events and numbers of patients with events were summarized. Time to occurrence of the primary efficacy and safety outcomes was summarized using Kaplan-Meier survival curves. Cox models were fitted to the time to primary efficacy outcome, primary safety outcome, both primary efficacy and safety outcomes and either primary efficacy or safety outcome and the differences between the Invasive and Medical Groups presented as hazard ratios (HRs) and corresponding 95% CIs. Descriptive statistics only were produced for the secondary outcomes because of the study being a pilot trial and not adequately powered for hypothesis testing for these outcomes.

### Ethics

The research study was reviewed and approved by the West of Scotland NHS Research Ethics Service (Reference 11-WS-0116).

### Trial Management

A Trial Management Group including the researchers and Local Principal Investigator on each of the 4 sites coordinated the study’s activities on a day-to-day basis.

The NHS Sponsor monitored the trial. Since the trial was a pilot, there was no Independent Data and Safety Monitoring Committee.^43^

## Results

Two hundred seventeen patients with an unplanned hospitalization for a confirmed or suspected NSTE-ACS and a history of prior CABG were screened during a 16-month period. The first patient was enrolled on February 20, 2012 (Figure 1; CONSORT diagram). Eighty-four (39%) of these patients were screen failures, including 24 (29%) who did not give consent and 60 (71%) who were ineligible (≥1 reason). The reasons for being ineligible included lack of a confirmed NSTE-ACS (n=42 [70%] patient), persisting unstable symptoms (n=39 [65%] patients), refractory ischemia (n=5 [8%] patients), unsuitable for invasive management (n=22 [37%] patients), no prior CABG (n=4 [7%] patients), and unable to provide informed consent (n=3 [5%] patients).

One hundred thirty-three (61%) subjects fulfilled eligibility criteria for the randomized trial (Figure 1) and 60 (mean±SD, 71±9 years of age, 43 [72%] male) were enrolled into the trial and randomized. Seventy-three (mean±SD, 72±10 years of age, 53 [73%] male) patients who were eligible for the trial were not randomized because of physician preference (n=58), patient preference (n=28), or both (n=15). The mean ages of the patients in the registry (72±10 years) and trial groups (71±9 years) were similar (*P*=0.46), as were the proportions of women (20 [27%] versus 17 [28%]; *P*=1.00).

### Baseline Characteristics

**Figure 2.**
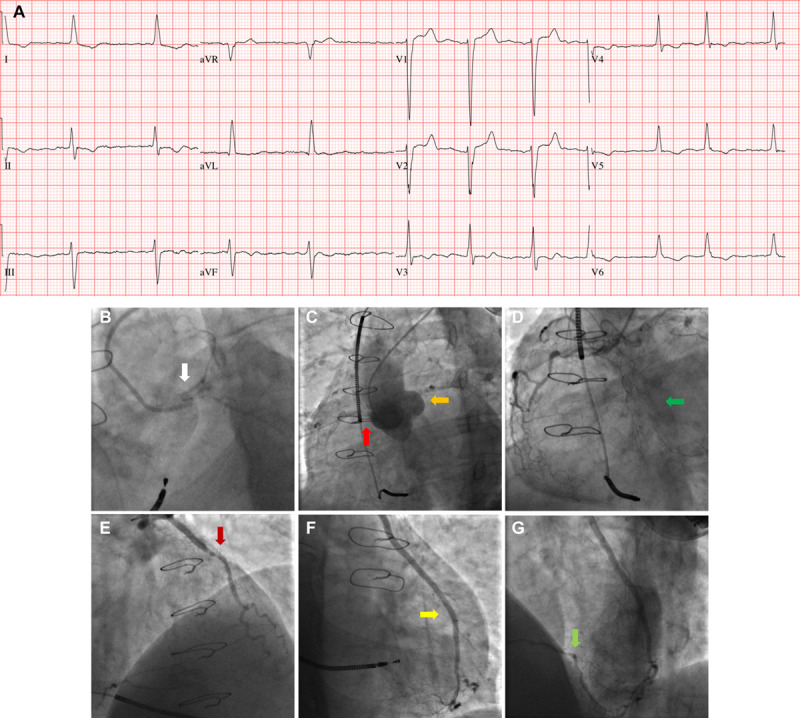
**Clinical case.** A 51-y-old male was hospitalized following an acute non-ST elevation acute coronary syndrome. **A**, Twelve-lead ECG demonstrated atrial fibrillation with ST depression and T-wave inversion in the lateral leads, which were not significantly changed from previous ECGs. The Global Registry of Acute Coronary Events score for death or myocardial infarction within 6 mo was 107. The past medical history included coronary artery bypass grafting 14 y previously, left ventricular systolic dysfunction, a cardiac defibrillator for primary prevention, and atrial fibrillation. The surgical record and graft history were not available. The day after admission to hospital, the patient provided written informed consent to participate in the CABG-ACS (coronary artery bypass graft acute coronary syndrome) trial, and he was randomized to the invasive group. Coronary angiography was performed on an urgent basis via the left radial artery. **B**, The native left main coronary artery was occluded at the ostium (white arrow). **C**, The saphenous vein grafts to the right coronary artery (RCA; red arrow) and obtuse marginal branch of the left coronary artery (orange arrow) were also occluded. **D**, Angiography of the native RCA revealed proximal and mid-vessel occlusions associated with bridging ipsilateral collateral connections (green arrow). **E**, The left internal mammary artery (LIMA) graft had a 70% to 80% stenosis (dark red arrow) involving the anastomosis with the left anterior descending (LAD) coronary artery with normal antegrade flow. This lesion was judged to be the culprit. The LIMA supplied collaterals to the distal branches of the RCA pointing to a large territory of jeopardized myocardium. Given the history of left ventricular dysfunction, the ischemic area-at-risk, and risks of percutaneous coronary intervention (PCI) to this stenosis, the treatment plan was for deferred management including uptitration of antiangina drug therapy and PCI to the LIMA should symptoms became refractory. **F**, Two mo later, the patient was readmitted because of persistent angina, and PCI to the insertional stenosis of the LIMA-LAD anastomosis stenosis was then performed (yellow arrow). Following predilatation, a 3.0×28 mm drug eluting stent was deployed at 17 atm. PCI was completed with high inflation postdilatation and an excellent final result was obtained. **G**, Angiography at the end of the procedure revealed antegrade filling of the distal LAD and retrograde filling of the posterior descending branch of the RCA via collateral connections from the LIMA-LAD system (light green arrow). Dual antiplatelet therapy was prescribed for 12 mo. The patient was hospitalized on 3 further occasions. He experienced a type 1 non-ST elevation myocardial infarction 4 mo later. In-stent restenosis was diagnosed and treated with additional PCI. Two mo later, he was then hospitalized with unstable angina and 2 mo after that he experienced another type 1 NSTEMI. He was medically managed on these occasions.

The characteristics of the trial participants are described in Table 2 and a clinical case is illustrated in Figure 2. The mean age was 71 years, and all of the participants had at least 1 concomitant health problem. Multimorbidity was very common (Table 2). Two thirds had a history of hypertension, one-third had diabetes mellitus, one-quarter had HF, and one-fifth had cerebrovascular disease or renal failure. The sample averages and rates of other comorbidities, age, sex, Charlson Comorbidity Index, Canadian Cardiovascular Society grade, frailty score, and medications were broadly similar between the groups. Fifty (83%) of the trial participants had a history of a left internal mammary artery graft.

**Table 2.**
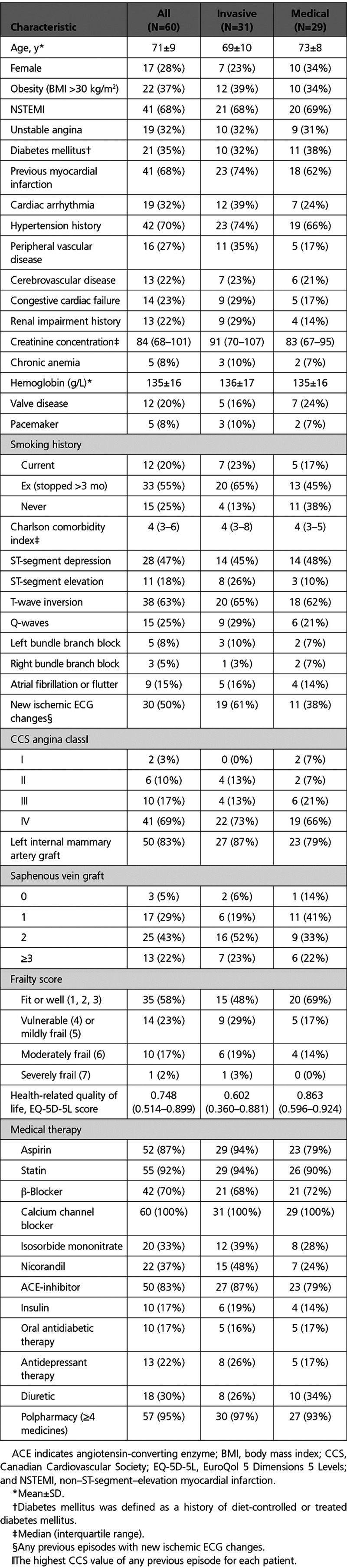
Baseline Clinical and Angiographic Characteristics of the Trial Participants

### Medical Therapy

Changes in secondary preventive medications and antianginal therapy during the index hospitalization were prescribed in the majority of participants, and the changes in medical therapy were similar in each group (Table 3).

**Table 3.**
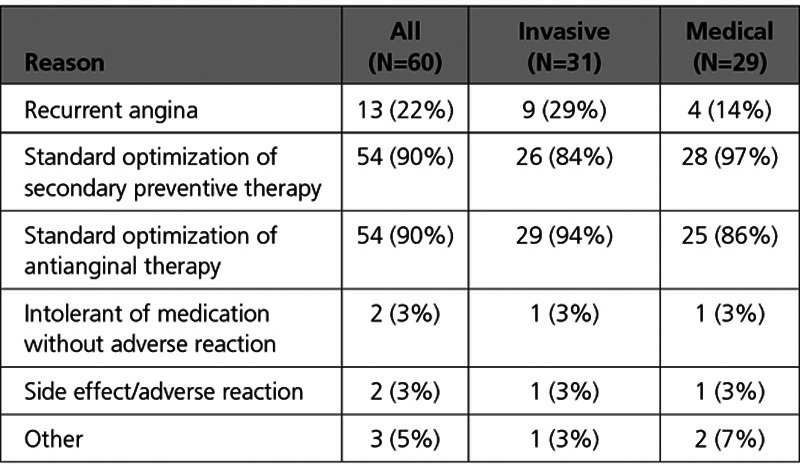
Reasons for Changing Medical Therapy During the Index Hospitalization

### Invasive Management

Invasive management was performed in all 31 participants in the invasive group (Table 4). Percutaneous coronary intervention was performed in 10 (32%) participants in the invasive group during the index hospitalization and 4 more patients in this group received PCI during a second procedure as part of a staged management plan (n=14 [45%], overall). The mean British Cardiovascular Intervention Society-1 Jeopardy Score at baseline in the Invasive Group pre- and post-PCI was 4.3±3.7 and 2.4±2.5, respectively, out of a possible maximum score of 12 (Table 4). During follow-up (≥18 months), 39 coronary angiogram procedures were performed in the invasive group (including 17 [44%] proceeding to PCI).

**Table 4.**
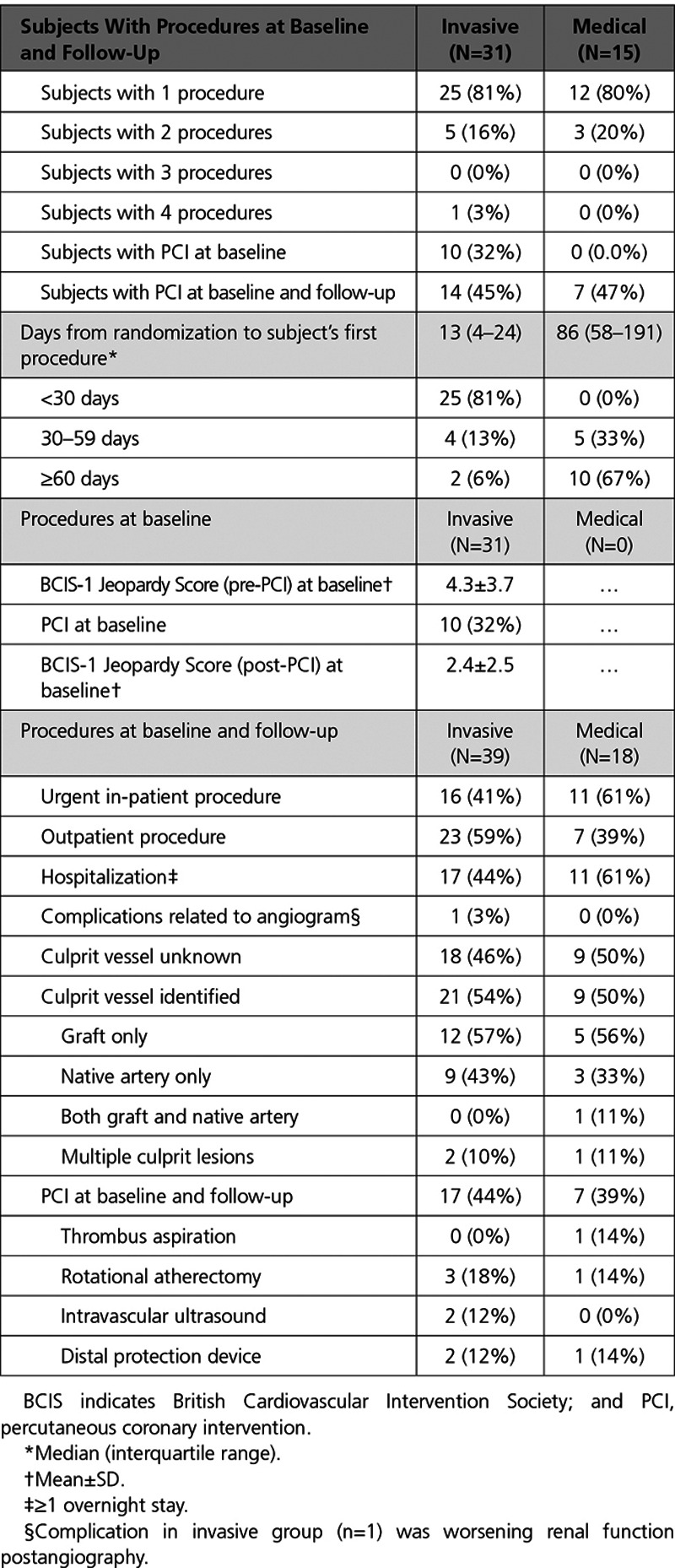
Invasive Procedures at Baseline (Index Admission) and Follow-Up (≥18 Months)

In the medical group, 1 male patient crossed over to invasive management on day 30 postrandomization because of recurrent angina. No revascularization targets were identified by coronary and graft angiography and medical management was adopted. Fifteen (52%) participants assigned to the medical group had an invasive procedure during the follow-up period, and 7 (47%) of these patients received PCI. Overall, 18 invasive procedures were performed in this group, and 7 (24%) patients were treated with PCI. None of the randomized patients received redo-CABG.

### Health Outcomes

During ≈2-years’ follow-up (median [interquartile range] 744 [570–853] days), the composite efficacy outcome of all-cause mortality, nonfatal MI, refractory ischemia, or HF hospitalization occurred in 13 (42%) participants in the invasive group and in 13 (45%) in the medical group (hazard ratio; 95% CI, 0.85 [0.39–1.83]; Table 5). Five participants died in the invasive group (2 cardiovascular, 2 noncardiovascular, and 1 unknown cause) and 3 died in the medical group (2 noncardiovascular and 1 unknown cause).

**Table 5.**
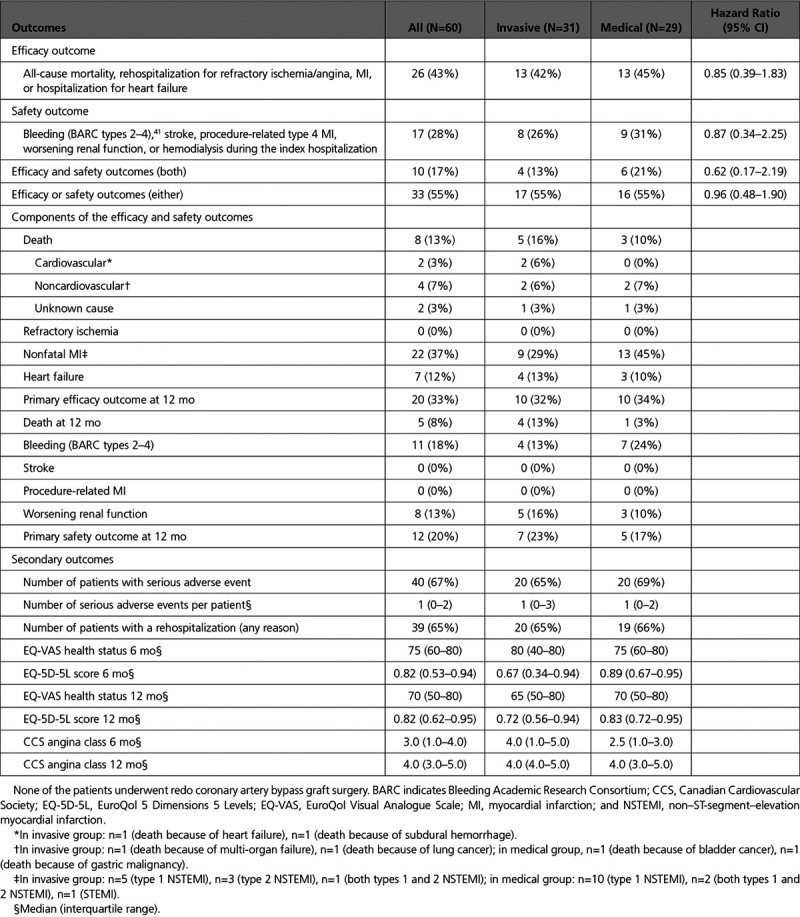
Primary and Secondary Outcomes Over Follow-Up Period (≥18 Months; Median 744 [Interquartile Range 570–853] D)

The composite safety outcome of major bleeding (Bleeding Academic Research Consortium types 2–4), stroke, procedure-related MI, or worsening renal function occurred in 8 (26%) participants in the invasive group and in 9 (31%) participants in the medical group (hazard ratio; 95% CI, 0.87 [0.34–2.25]; Table 5). Bleeding occurred in 4 (13%) and 7 (24%) patients in the invasive and medical groups, respectively. Worsening renal function occurred in 5 (16%) patients in the Invasive Group compared with 3 (10%) patients in the Medical Group.

**Figure 3.**
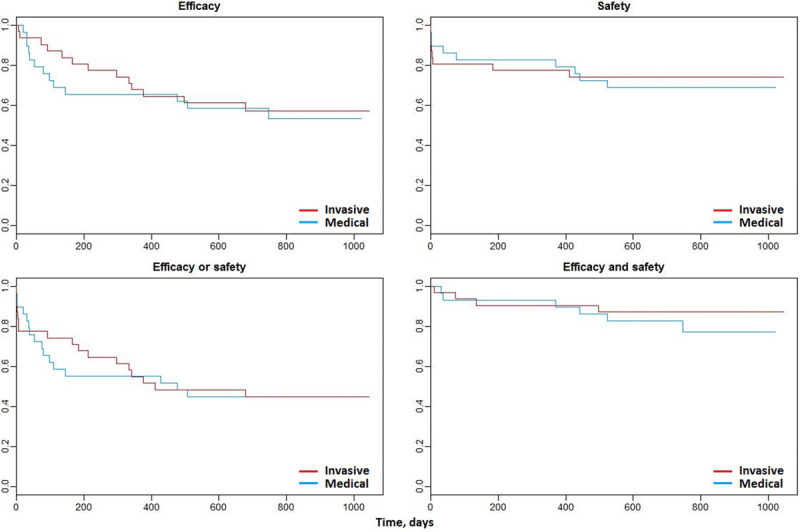
**Kaplan-Meier survival curves for time to occurrence of the composite outcomes for efficacy and safety, by study group.**

Overall, 33 (55%) participants experienced at least one of these events: 17 (55%) of 31 participants in the invasive group and 16 (55%) of participants in the medical group (hazard ratio; 95% CI, 0.96 (0.48–1.90). The Kaplan-Meier survival curves are shown in Figure 3. Overall, there were no differences between the groups. The time course for efficacy events appeared to differ between the groups with proportionately more events occurring earlier in the medical group and proportionately more events occurring later in the invasive group.

### Health Status

Compared with the medical group, the invasive group had higher degree of impairment in health-related quality of life (eg, lower EuroQol 5 Dimensions 5 Levels score) at baseline (Table 2) and 6 months, but by 12 months, the average group scores were similar (Table 5).

### Angina

Functional limitation from angina, reflected by the Canadian Cardiovascular Society angina class, was similar between the groups at 6 and 12 months.

## Discussion

This study informs the evidence gap relating to invasive versus medical management in patients presenting with an acute NSTE-ACS and prior CABG. We report the first randomized, controlled, multicenter, trial of invasive and noninvasive management strategies in this patient group. The main findings are (1) enrollment into the randomized trial was feasible but challenging (Figure 1; CONSORT diagram). The age and sex distributions of the patients in the trial and registry groups were similar, suggesting minimal selection bias; (2) adherence to the randomized strategy within the 30 day crossover period was achieved in all but one of the participants; (3) revascularization was initially performed in only one-third of the invasive group; (4) the majority of the trial population experienced a major adverse event during follow-up; and (5) no between-group differences in these events, but the trial was not powered for these. The trial provides proof-of-concept evidence that an initial noninvasive management strategy in NSTE-ACS patients with prior CABG is feasible. Importantly, the results support the rationale for a substantive health outcome trial of these strategies in this patient group.

Patients with a prior CABG who present with an acute coronary syndrome have usually experienced chronic myocardial ischemia for years. In this study, 68% of participants had a prior MI. The cause of an NSTE-ACS in patients with a prior CABG may be the eventual occlusion of a chronically diseased graft, a mismatch in myocardial blood supply:demand (type 2 MI) whereby collateral blood supply fails to meet myocardial demand or occlusion of a native coronary artery. The clinical case presented in our study (Figure 2) is 1 example. Chronic ischemia stimulates arteriogenesis promoting coronary collateral connections to deliver oxygenated blood to ischemic myocardium.^44^ These microconnections may be extensive and imperceptible at angiography.

In our study, a culprit vessel was only identified by the attending cardiologist in half of the invasively managed patients. This conundrum reflects the diagnostic uncertainties associated with complex, multivessel native coronary and bypass graft disease. PCI was initially performed in one-third of the invasive group. This may reflect uncertainties about performing complex PCI when the culprit lesion is not obvious and when procedural risks may be felt to be high. Further, multimorbidity may limit the potential for revascularization to improve quality of life. Finally, the overall risk:benefit ratio of performing PCI in this population may also be influenced by the fact that the participants had stabilized with medical therapy. PCI was only performed in a minority of the invasive group.

### Burden of Disease

In contemporary trials involving NSTE-ACS patients, the 12-month major adverse cardiac event rate is usually 8% to 10%. In our trial, the rate was over 4× higher (45% met either primary efficacy or safety outcomes at 12 months), increasing to 55% overall. The rising event rate over time contrasts with other trials in NSTE-ACS populations in which major adverse cardiac event rates tend to plateau during the first 3 months post-MI. The older age and universal presence of multimorbidity probably explain the differences in prognosis between NSTE-ACS patients with versus without prior CABG. Our results support the hypothesis that routine noninvasive management could be initially adopted for patients with an NSTE-ACS and prior CABG except in the minority with ongoing ischemia.

### Advances in Interventional Management

In recent years, radial artery access has become the standard approach for invasive management rather than femoral artery access. The left radial artery allows access in patients with a left internal mammary artery graft. Adoption of advanced techniques for revascularization of chronic native vessel occlusive disease might lead to higher rates of successful revascularization.^45^ This possibility could be prospectively assessed in a larger multicenter trial.

### Noninvasive Imaging

Functional imaging to elicit inducible ischemia, notably with stress cardiovascular magnetic resonance, echocardiography, or myocardial perfusion scintigraphy, may be useful. However, these tests may be logistically challenging to perform on an emergent basis. Computerized tomography coronary angiography is useful for imaging grafts but not necessarily for imaging native coronary arteries because calcification provokes artifacts, reducing diagnostic accuracy. For this reason, in our opinion, computerized tomography coronary angiography has limited clinical utility to provide a comprehensive diagnostic evaluation in this patient population.

### Future Substantive Trial of Invasive Versus Noninvasive Management in Patients With a NSTE-ACS and Prior CABG

Some of the previous pivotal trials excluded patients with prior CABG (Table 1). The reasons for excluding these patients may be because of their distinct complexities relating to occlusive native vessel coronary artery disease, graft disease, and concomitant health problems. Consequently, practice guidelines are not evidence based in this subgroup meaning that clinicians lack relevant information to inform decision-making.

One of the primary aims of our pilot trial was to provide information on whether a larger trial in this NSTE-ACS subgroup might be feasible. Adherence to the randomized strategy was achieved in all but one of the participants, indicating that the interventions were feasible. Half of the medical group subjects underwent invasive angiography during follow-up but only a minority (24%) received PCI.

This study was logistically challenging to deliver. First, the grant committee raised concern about the ethics of randomizing study participants to noninvasive management and rejected our application for funding. The results of our trial provide reassurance in this regard. Without core funding support, this study was all the more difficult to deliver. Screening and enrollment were time consuming. The population mainly included frail and elderly participants (mean age, 71±9 years; Table 2). Physician preference was a determining factor for enrollment. Over half of the patients screened were deemed ineligible for randomization based on physician preference. Our experience indicates that a multicenter phase 3 trial will present logistical challenges. To deliver that trial, support from physicians during urgent care will be needed. To that end, we hope that the preliminary evidence of similar adverse event rates between the groups will give physicians and patients confidence to participate. We envisage the future trial would be pragmatic, with an all-comers approach to enrollment and eligibility criteria similar to the pilot. We envisage a noninferiority design for invasive versus noninvasive management and a primary composite outcome that includes all-cause mortality and spontaneous adverse events that are not determined by clinicians’ decisions to minimize bias. The trial will champion advanced interventional techniques for native vessel revascularization. If the noninferiority hypothesis is confirmed, then noninvasive management could be considered a default standard of care for medically stabilized patients.

### Potential Impact of a Future Trial

About 1 in 10 NSTE-ACS patients have prior CABG. This rate is likely to remain stable in the coming years reflecting sustained referrals for CABG in the past decade and increasing longevity. Our results support the hypothesis that a noninvasive strategy could be initially adopted for most NSTE-ACS patients with prior CABG, reserving invasive management for patients with persistent or recurrent, ischemia. The results from a future phase 3/4 trial could be implemented in daily practice, potentially reducing variations in management, enabling more efficient resource utilization, and allowing NSTE-ACS patients with prior CABG to reach critical points in the care pathway more quickly.

### Limitations

The sample size is insufficient to draw conclusions about the effectiveness of the clinical strategies. The study predates recent advances in interventional techniques for chronic occlusive coronary artery disease.

### Conclusions

In a pilot study, we observed no difference in clinical outcomes between patients with NSTE-ACS and prior CABG undergoing either noninvasive or routine invasive management. A randomized trial of these strategies is feasible. A substantive trial involving contemporary invasive and medical therapies seems warranted.

## Acknowledgments

M.M.Y. Lee coordinated study, enrolled patients, and collected clinical data. M.C. Petrie, Drs Rocchiccioli, Simpson, and Jackson participated Clinical Event Committee. Drs Rae, Berry, Findlay, and Balachandran were local Principal Investigators. A. Brown, Dr McEntegart, A. Shaukat, Dr Hood, Dr Peat, Dr Murphy, A.J. Cormack, and N.B. Bukov enrolled patients and collected clinical data. Drs Corcoran and Mangion collected clinical data. Dr Cialdella and N.P. Sidik performed angiographic analyses for British Cardiovascular Intervention Society-1 Jeopardy Score. K.G. Oldroyd performed critical appraisal of manuscript. Dr Wu participated in study design and health economics. Drs Ford, McConnachie, and Barry participated in biostatistics. Dr Berry is a Chief Investigator.

## Sources of Funding

Dr Berry was supported by a Scottish Funding Council Senior Clinical Fellowship and a British Heart Foundation (BHF) Centre of Research Excellence Award (RE/13/5/30177; RE/18/6134217). Drs. Mangion and Sidik were supported by BHF Clinical Training Fellowships (FS/15/54/31639; FS/17/26/32744).

## Disclosures

Based on institutional contracts with the University of Glasgow, Dr Berry has held research and consultancy agreements with Abbott Vascular, AstraZeneca, Boehringer Ingelheim, HeartFlow, GSK, Novartis, Philips, and Siemens Healthcare. The other authors report no conflicts.
